# Evaluation of the humoral and mucosal immune response of a multiepitope vaccine against COVID-19 in pigs

**DOI:** 10.3389/fimmu.2023.1276950

**Published:** 2023-12-20

**Authors:** Juan Mosqueda, Diego Josimar Hernández-Silva, Marco Antonio Vega-López, Lineth J. Vega-Rojas, Rolando Beltrán, Andrés Velasco-Elizondo, María del Carmen Ramírez-Estudillo, Mario Fragoso-Saavedra, Chyntia Pérez-Almeida, Jesús Hernández, Edgar A. Melgoza-González, Diana Hinojosa-Trujillo, Miguel Ángel Mercado-Uriostegui, Alma Susana Mejía-López, Carlos Rivera-Ballesteros, Teresa García-Gasca

**Affiliations:** ^1^ Immunology and Vaccines Laboratory, Facultad de Ciencias Naturales, Universidad Autonoma de Queretaro, Carretera a Chichimequillas, Santiago de Querétaro, Querétaro, Mexico; ^2^ Centro de Investigación y de Estudios Avanzados (CINVESTAV) del Instituto Politécnico Nacional, Departamento de Infectómica y Patogénesis Molecular, Laboratorio de Inmunobiología de las Mucosas, Ciudad de México, Mexico; ^3^ Facultad de Medicina Veterinaria y Zootecnia, Universidad Nacional Autónoma de México, Ciudad de México, Mexico; ^4^ Laboratorio de Inmunología, Centro de Investigación en Alimentación y Desarrollo, A.C, Hermosillo, Mexico; ^5^ Facultad de Ciencias Naturales, Universidad Autónoma de Querétaro, Querétaro, Mexico

**Keywords:** COVID-19, recombinant protein, multiepitopic vaccine, SARS-CoV-2, humoral response, mucosal immunity

## Abstract

**Introduction:**

This study evaluated the immune response to a multiepitope recombinant chimeric protein (CHIVAX) containing B- and T-cell epitopes of the SARS-CoV-2 spike’s receptor binding domain (RBD) in a translational porcine model for pre-clinical studies.

**Methods:**

We generated a multiepitope recombinant protein engineered to include six coding conserved epitopes from the RBD domain of the SARS-CoV-2 S protein. Pigs were divided into groups and immunized with different doses of the protein, with serum samples collected over time to determine antibody responses by indirect ELISA and antibody titration. Peptide recognition was also analyzed by Western blotting. A surrogate neutralization assay with recombinant ACE2 and RBDs was performed. Intranasal doses of the immunogen were also prepared and tested on Vietnamese minipigs.

**Results:**

When the immunogen was administered subcutaneously, it induced specific IgG antibodies in pigs, and higher doses correlated with higher antibody levels. Antibodies from immunized pigs recognized individual peptides in the multiepitope vaccine and inhibited RBD-ACE2 binding for five variants of concern (VOC). Comparative antigen delivery methods showed that both, subcutaneous and combined subcutaneous/intranasal approaches, induced specific IgG and IgA antibodies, with the subcutaneous approach having superior neutralizing activity. CHIVAX elicited systemic immunity, evidenced by specific IgG antibodies in the serum, and local mucosal immunity, indicated by IgA antibodies in saliva, nasal, and bronchoalveolar lavage secretions. Importantly, these antibodies demonstrated neutralizing activity against SARS-CoV-2 *in vitro*.

**Discussion:**

The elicited antibodies recognized individual epitopes on the chimeric protein and demonstrated the capacity to block RBD-ACE2 binding of the ancestral SARS-CoV-2 strain and four VOCs. The findings provide proof of concept for using multiepitope recombinant antigens and a combined immunization protocol to induce a neutralizing immune response against SARS-CoV-2 in the pig translational model for preclinical studies.

## 1 Introduction

COVID-19, caused by the SARS-CoV-2 virus, first appeared in Wuhan, China, at the end of 2019 (1). To date, this disease has achieved sustained person-to-person transmission as well as infections in some animal species, which is why it was declared a pandemic by the WHO on March 11, 2020 (2). Vaccines are one of the most effective methods to control diseases, including COVID-19. Currently, there are vaccines against this disease that have already been authorized for full use in the population, including vaccines based on messenger RNA, adenoviral vectors or inactivated viruses (3–5). Few licensed vaccines that are based on recombinant proteins (6). It had been anticipated that the demand for these vaccines would be so high that vaccines would not be produced for all countries. More vaccines against COVID-19 and other emerging and re-emerging diseases are needed. One strategy that is feasible and easy to implement is the development of recombinant protein-based vaccines. Vaccines based on proteins have a long history of success and have some advantages over nucleic acid and viral vector vaccines, such as low distribution and storage costs, and they can be applied more than twice without causing an immune response against the viral vector (7, 8). Furthermore, it is possible to modify the structure of the vaccine as the pathogen evolves making updated immune responses easily achieved. However, the selection of antigens is very important because vaccines based on a single antigen are poorly immunogenic, as they generate an immune response against a few epitopes. One solution to this problem is to identify B- and T-cell epitopes that induce the production of neutralizing antibodies and incorporate and assemble them into a single synthetic gene that encodes a multiepitopic molecule. Our group has previously shown that peptides with B- and T-cell epitopes can be identified using an initial bioinformatics strategy and subsequently perform an evaluation of the immunogenicity of each of the epitope-containing peptides (9–11). We hypothesize that the genetic sequences of those epitopes that generate antibodies can be incorporated into this multiepitope vaccine, also known as a chimera. The spike (S) protein of SARS viruses has a receptor binding domain (RBD) that allows binding to angiotensin converting enzyme 2 (ACE2) to initiate the invasion process into cells (12). Protein S is the leading candidate in the development of vaccines against this disease, and it has been shown that antibodies against RBD are sufficient to block invasion (13). Moreover, it has been demonstrated that variant of concern (VOC) cross-reactive antibodies are generated only to the RBD domain of the S protein, which motivates the development of new RBD-based vaccines against SARS-CoV-2 (14).

On the other hand, most of the vaccines available are applied by the intramuscular (IM) route, which is very effective in eliciting a systemic immune response but does not necessarily protect the mucosae (15, 16), the main gate of entrance for pathogens (17, 18). This type of response may allow the infection of the mucosae without causing symptoms of the disease but releasing the pathogen into the environment (healthy carrier). Mucosal vaccination has the potential to prevent infection and avoid pathogen transmission (19, 20) through the local production of neutralizing antibodies (nAbs), especially secretory IgA (SIgA), and the generation of resident memory T and B lymphocytes (21–24). To achieve this, the antigen (Ag) must reach the mucosal inductive sites to avoid tolerance induction (25, 26). However, direct mucosal Ag administration cannot ensure that effective doses reach these inductive sites, it may induce tolerance, and it requires aggressive adjuvants, which prevent its use in human and animal prophylaxis. Using a combined immunization protocol (parenteral and mucosal), it is possible to circumvent these problems by eliciting an integral (serum and mucosal) antibody response (27, 28).

In this work, we report the evaluation of humoral and mucosal immune responses in pigs to a vaccine based on a multiepitopic recombinant chimeric protein (CHIVAX) containing B- and T-cell epitopes of the SARS-CoV-2 spike RBD. The pig immunome is more similar to that of the human compared to that of the mouse; pigs must therefore be considered a highly relevant model when studying human immune activation. The difference in mouse body size and metabolism makes studies on the dose effect of adjuvants and peptides impossible to extrapolate to human vaccine formulation (29, 30). The aims of this work were the proof of concept of using multiepitope recombinant antigens in a combined immunization protocol to obtain an Ab response in serum and mucosal secretions [saliva, nasal and bronchoalveolar lavage (BAL)] with neutralizing activity against the SARS-CoV-2 virus, in a translational porcine model of research useful for the pre-clinical phase of future vaccines.

## 2 Materials and methods

### 2.1 Ethics statement

All animal procedures in this study were performed following a protocol reviewed and approved by the Research Ethics Committee of the Autonomous University of Queretaro (DIP/580-2020) and the Institutional Animal Care and Use Committee (0315–21) of CINVESTAV, following the Mexican Official Norm NOM-062-ZOO-1999.

### 2.2 Construction and synthetic gene design

The SARS-CoV-2 S protein sequences from the NCBI Database published on April 6^th^, 2020, were used to find immunogenic peptides, and design a chimeric protein. Spike protein sequences were subjected to analysis by bioinformatics algorithms to determine the following characteristics (each characteristic is described with the bioinformatics tools that were used): the Conserved Domain Database from NCBI (available at https://www.ncbi.nlm.nih.gov/cdd/) (31) and the Simple Modular Architecture Research Tool (SMART, available at http://smart.embl-heidelberg.de/) (32, 33) were used to localize the RBD domain map in the spike protein, used by coronaviruses to enter the host cell (34). Protein hydrophobicity was determined with the Protscale tool (available at https://web.expasy.org/protscale/) (35). Signal peptide presence was determined with SignalIP 5.0 (available at https://services.healthtech.dtu.dk/service.php?SignalP-5.0) (36). Protein transmembrane regions were predicted by the TMHMM 2.0 tool (available at https://services.healthtech.dtu.dk/service.php?TMHMM-2.0) (37). Thereafter, conserved peptides in RBD sequences in different SARS-CoV-2 variants were resolved by multiple sequence alignment using MUSCLE (https://www.ebi.ac.uk/Tools/msa/muscle/) (38). Subsequently, B and T epitope predictions in the conserved peptides were carried out using ABCPred (available at https://webs.iiitd.edu.in/raghava/abcpred/ABC_submission.html) (39), BCEPred (available at https://webs.iiitd.edu.in/raghava/bcepred/index.html) (40), and the Immune Epitope database (available at https://www.iedb.org/) (41).

A multiepitope recombinant protein (CHIVAX) was designed by selecting six peptides located in the RBD sequence. These peptides were selected by first identifying B- and T-cell epitopes that were conserved on all the RBD sequences published at that time (March 2020). Once each immunogenic peptide was selected, a multiepitopic sequence was designed in such a way that the peptides were organized into a single amino acid primary structure to be consistent with the order in the original RBD sequence. Several versions of the random multiepitopic sequences were analyzed using the same epitope prediction algorithms mentioned above to determine the presence of the originally predicted epitopes and the prediction of new juxtaposed epitopes formed within two adjacent peptides. When juxtaposed epitopes were predicted, some amino acids were added between peptides as spacers or peptides were moved to another part of the sequence to avoid the formation of new, nonrelevant epitopes. Reverse translation of the amino acid sequence and preferential codon usage for the *Escherichia coli* B strain was carried out by the Codon Optimization OnLine tool (no longer available on the internet) (42). Once the nucleotide sequence was obtained, a synthetic DNA sequence of the chimeric gene cloned in vector pUC57 was commercially acquired (Bio Basic Inc., Ontario, Canada). Additionally, the peptides were commercially synthesized in both linear and 8-branch Multi-Antigen Peptide (MASP-8) forms.

### 2.3 Gene cloning, subcloning and sequencing

The pUC57 vector containing the synthetic chimera gene was used as a template to produce amplicon copies by PCR using a high-fidelity DNA polymerase (Accuzyme DNA polymerase, Meridian Bioscience), and proper amplification was confirmed by 1% agarose gel electrophoresis. Thereafter, amplicons were purified by a Spin Column DNA Gel Extraction Kit (Bio Basic Inc, Ontario, Canada) to discard amplification reaction contaminants. The pENTR-D/TOPO vector was mixed with purified amplicons following the manufacturer’s instructions (Invitrogen cat. K240020, Massachusetts, USA). One Shot TOP 10 chemically competent *E. coli* cells were transformed, and proper cloning was confirmed by PCR with specific primers. The entry clone described above was used to transfer the gene into the pDEST-17 expression vector by Gateway technology. The cloned product was used to transform DH5 alpha high-efficiency library chemically competent cells following the manufacturer’s instructions (Invitrogen, Massachusetts, USA). Proper cloning was confirmed by PCR using T7 promoter-specific forward and reverse primers and by commercial sequencing at Laboratorio de Servicios Genomicos (LANGEBIO, Guanajuato, Mexico). Finally, BL-21-AI chemically competent *E. coli* cells were transformed using the expression vector obtained previously.

### 2.4 Protein expression and protein identification by immunodetection

Four BL-21-AI (Invitrogen, Cat# C607003)-transformed clones were randomly chosen to induce protein expression. Clones’ pre-inoculum was grown in LB media with 100 μg/mL ampicillin overnight and was used to initiate a fresh culture starting with 0.05 O.D. λ600 until the culture reached 0.4 O.D. λ600. The culture was split into two equal volumes, and in one volume, L-arabinose was added as an expression inducer. Both cultures were incubated for approximately 4 hours, and samples were taken prior to expression induction and every hour for four hours. A control culture of non-transformed BL-21-AI *E. coli* cells was carried out using the same procedure and incubation conditions. To confirm the expression of the cloned gene, Western blot immunodetection was performed. Proteins were separated by acrylamide gel electrophoresis in denaturing conditions using induced cell lysates. Proteins were transferred onto nitrocellulose membranes. One membrane was incubated with a commercial mouse monoclonal anti-6x-His-Tag antibody (Cat #MA-21315 Invitrogen, California, USA) at a 1:10,000 dilution for one hour at room temperature. Another membrane was incubated with serum from a positive patient diagnosed with COVID-19 by qPCR and collected approximately 3 weeks after clinical signs and symptoms disappeared. Another human serum collected before the pandemic began was used as a negative control.

### 2.5 Bioreactor culture scaling

BL-21-AI *E. coli* clones described previously were used to scale up chimeric protein production in a batch fermentation system composed of a stirred glass tank UniVessel® with the control tower Biostat® A, Sartorius. LB miller (Merck, Millipore) supplemented with 100 µg/mL ampicillin was used as the culture medium, and fermentation conditions were established at 37°C, pH 6.8, and 40% dissolved oxygen. An overnight culture of chimeric protein transformant *E. coli* was used as inoculum by adjusting the volume to start fermentation with 0.5 O.D. at λ600 nm. When a 0.4 O.D. at λ600 nm, protein expression was induced by adding L-arabinose at a final concentration of 0.2%. Fermentation conditions were maintained, and 1 mL samples were taken every hour to measure optical density. Fermentation was stopped when the culture reached 1.2 O.D. λ600 nm. Subsequently, all biomass was harvested by a centrifugation pulse at 12,000 rpm, and biomass pellets were stored at -20°C for the subsequent protein purification process.

### 2.6 Protein purification

For recombinant protein purification, ion metallic affinity chromatography was performed using nickel gravity columns under denaturing conditions following the manufacturer’s recommendation with modifications (Ni-NTA Superflow Columns, Cat. 30622, Qiagen, Hilden, Germany). Expression-induced cultured cells were pelleted by centrifugation at 1,500 x g and resuspended in 5 mL phosphate buffer (NaCl 137 mM, KCl 2.7 mM, Na_2_HPO_4_ 10 mM, KH_2_PO_4_ 1.8 mM pH 7.4). Subsequently, cell lysis was performed by sonication using 10 second pulses at 80% amplitude in an ice bath. The cell lysate was washed by three cycles of centrifugation at 18,000×g with 5 mL phosphate buffer. The washed pellet was resuspended in chromatography binding buffer (100 mM, 10 mM Tris, 8 M urea, pH 8.0) and homogenized for 1 hour with mild orbital agitation. The protein suspension was filtered with 1.2-micron membranes and then loaded into the column. To allow protein binding with the resin, the column was incubated for 1 hour and mixed at a very slow speed. Thereafter, the resin was washed with 4 mL of washing solution (same as binding buffer with 20 mM imidazole and pH 6.3) and mixed for 25 minutes. Elution was performed by adding 1 mL elution buffer (same as binding buffer pH 5.9). Eight fractions were collected, and a second elution round was performed using the same buffer with a pH of 4.5. Ten fractions were collected. Fractions were dialyzed using a regenerated cellulose membrane in phosphate buffer at pH 7.4. All incubations during purification and dialysis were performed at room temperature. All fractions were analyzed by SDS-PAGE, and the protein concentration was determined by Bradford assay.

### 2.7 Vaccine dose and route of administration

#### 2.7.1 Subcutaneous doses

Subcutaneous (SC) doses were prepared using three different protein quantities: 30, 60 and 100 μg. Protein was mixed with sterile phosphate buffer and Montanide ISA 71 vg (Seppic, Courbevoie, France) as an adjuvant at a 1:1 ratio. The formulation was carried out by sonication with 5-minute pulses at 100% amplitude in an ice bath with cooling steps. In every cooling step, 1 μL of sample was analyzed under an optical microscope with a 100 × amplification objective. The formed microemulsion was confirmed by observation of a homogeneous micelle less than or equal to 2 μm size. The microemulsion was aliquoted in 1 mL doses and kept at 4°C until use.

#### 2.7.2 Subcutaneous immunization

**Figure 1:**
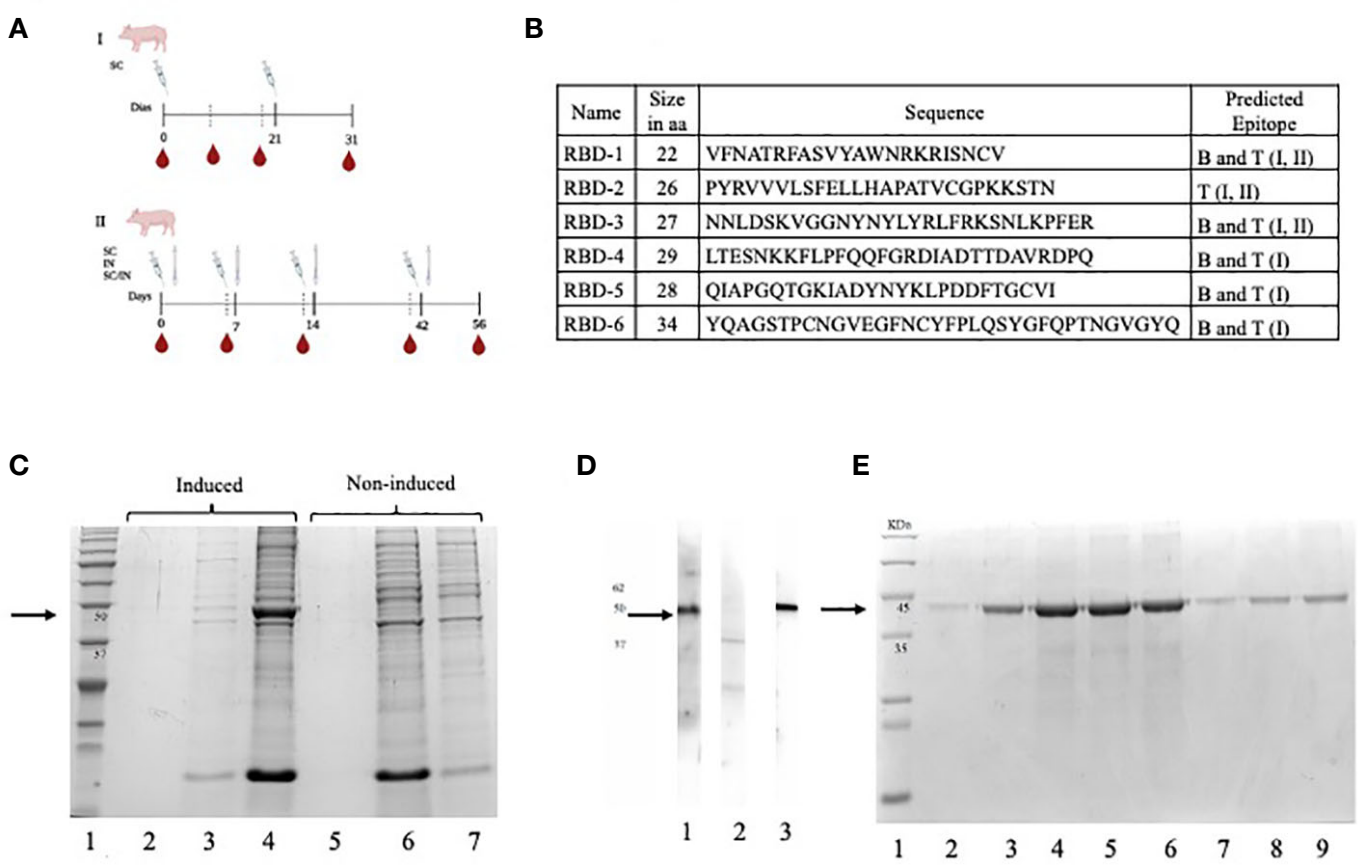
**(A)** Immunization schemes for two independent experiments, the first using subcutaneous (SC) (I) route and the second using SC, IN, and a combined SC/IN protocol (II) **(B)** Selected peptides that make up the multi-epitope vaccine (CHIVAX). Name, size, sequence, and type of epitope predicted in the designed RBD peptides. **(C)** A polyacrylamide gel was loaded with the induced culture medium (lane 2), induced *E coli* soluble protein lysate fraction (lane 3), induced *E coli* lysate insoluble fraction (lane 4), non-induced culture medium (lane 5), non-induced *E coli* lysate soluble protein fraction (lane 6) and non-induced *E coli* lysate insoluble fraction (lane 7). **(D)** The recombinant protein was loaded onto a polyacrylamide gel, transferred to a nitrocellulose membrane, and subsequently divided into 3 membranes. The serum sample from a COVID-19-positive human was incubated with the first membrane (lane 1), the serum sample from a COVID-19-negative human was incubated with the second membrane (lane 2), and the control monoclonal anti-His tag antibody was incubated with the third membrane (lane 3). These samples were employed to detect the recombinant protein on the membrane. **(E)** The purified and dialyzed protein fractions were loaded onto a polyacrylamide gel under denaturing conditions for subsequent analysis by SDS-PAGE. The protein fractions ranging from the second to the eightieth were loaded onto the gel individually in lanes 2 to 9. The black arrows indicate the molecular size of target protein (48 KDa).

To test the recombinant protein as an immunogen, an immunization protocol was performed in pigs. For this, twenty, eight-week-old commercial Yorkshire male and female pigs were separated into four groups of five animals each. One group received 30 μg of protein, the second group received 60 μg, and a third group received 100 μg of protein. The fourth group received only adjuvant as a control. Each animal in each group received two subcutaneous (SC) antigen doses at 21-day intervals. Blood was collected without anticoagulant at days 0, 10, 21, and 31 (for protocol, see **Figure 1A**). Serum was separated and stored at -21°C until use.

### 2.8 Evaluation of the humoral immune response

Antibodies present in the serum were determined by indirect ELISA (iELISA) with the chimeric protein and with anti-pig IgG (H + L) conjugated with horseradish peroxidase (Bethyl, Montgomery, Texas, USA). The plates were coated with 4 µg/mL of the protein (100 µL/well). Then, they were incubated at 4°C overnight. The plates were washed 3 times with 1× Phosphate Buffered Saline with 0.05% Tween-20 (PBS-T). After that, they were blocked with 5% skimmed milk (Svelty-Nestle), allowed to incubate at 37°C for 1 hour and washed 3 times. One hundred microliters of serum diluted 1:2000 from days 0, 10, 21 and 31 post immunization were added. The plates were incubated at 37°C for 1 hour on a rotatory shaker at 200 rpm and washed 3 times. One hundred microliters per well of anti-pig IgG at a 1:200,000 dilution was added and incubated at 37°C for 45 min at 200 rpm, and the washing was repeated. OPD substrate (Sigma Aldrich, Germany) was added and incubated for 25 min, and the optical density (O. D.) was measured at 450 nm. Statistical significance was tested by Tukey’s multivariable comparison test α=0.05.

### 2.9 Antibody titration

Antibody titers present in sera of immunized pigs were determined by iELISA with the chimeric protein and horseradish peroxidase-conjugated anti-pig IgG (H + L) (Bethyl, Montgomery, Texas, USA). ELISA plates were coated overnight at 4°C with 4 µg/mL recombinant protein (100 µL per well) in bicarbonate buffer, and the plates were washed 3 times with PBS-T. The plates were blocked with PBS-T containing 5% skimmed milk (Svelty-Nestlé) for 1 h at 37°C and 200 rpm and washed 3 times. A total of 100 µL of each serum was added to the plates from days 0 and 31 after immunization at serial dilutions from 1:2,000 to 1:4,096,000. The plates were incubated at 37°C for 1 h and 200 rpm and washed 3 times. A 1:200,000 diluted anti-pig IgG (100 µL per well) was added and incubated for 45 min at 37°C and 200 rpm, and the plates were washed three more times. OPD substrate (Sigma Aldrich, Germany) was added and incubated for 25 min, and the optical density (O. D.) was measured at 450 nm. Statistical significance was tested by Tukey’s multivariable comparison test, α=0.05.

### 2.10 Recognition of individual peptides

To test whether the sera of the animals (per group) had specific antibodies that recognized each of the peptides that were included in the chimera protein, an indirect ELISA was performed. For this, a mixture of the sera of the animals of each group and anti-Pig IgG conjugated with horseradish peroxidase (Bethyl, Montgomery, Texas, USA) were used. First, the plates were coated with 0.01 mg/mL of each peptide separately (100 µL/well) and incubated at 4°C overnight. Then, the plates were washed 3 times with 0.05% PBS-T. They were blocked with 5% skimmed milk (Svelty-Nestle) and allowed to incubate at 37°C for 1 hour at 200 rpm, and the washes were repeated. Sera from days 0 and 31 post immunization from each group were pooled in 1:80 dilutions, and 100 µL per well of serum from each group was added. The plates were incubated at 37°C for 1 hour at 200 rpm, and the washes were repeated. One hundred microliters per well of anti-pig IgG at a 1:200,000 dilution was added and incubated at 37°C for 45 min at 200 rpm, and the washes were repeated. Finally, OPD as substrate (Sigma Aldrich, Germany) was added and allowed to incubate for 25 minutes, and the optical density (O. D.) was obtained. The plates were read on an ELISA microplate reader (Bio-Rad, Hercules, CA, USA) at 450 nm. Each serum sample was analyzed in triplicate. The cutoff points were calculated using the mean O.I. D values, and statistical significance was tested by nonpaired multiple T tests with the Holm-Sidak method.

### 2.11 Production of recombinant ACE_2_, RBDs and surrogate neutralization assay for SARS-CoV-2

The coding sequences of hACE_2_ and the SARS-CoV-2 RBD were optimized for a mammalian expression system, synthesized, and cloned into a pcDNA3.1 (–) vector by GenScript (GenScript, New Jersey, USA). To produce the RBDs of VOC, site-directed mutagenesis was performed. The expression gene construct was preceded by a signal peptide and a Hist-tag (6xHist) terminal carboxyl domain as previously described (43). A surrogate neutralization assay was performed as previously described (44), with modifications. Each plate included two controls: one positive control (pooled from SARS-CoV-2-infected people) and one negative control (pooled from SARS-CoV-2-negative people) in duplicate. The results were expressed as the percentage of hACE_2_-RBD binding inhibition.

### 2.12 Intranasal doses

To test the recombinant protein as an intranasal (IN) immunogen, doses containing 200 μg of the antigen in sterile phosphate buffer without adjuvant were prepared following a previously reported protocol (45). Briefly, doses containing 200 μg of the antigen in sterile phosphate buffer without adjuvant were prepared. For subcutaneous (SC) immunizations, doses were prepared using 100 μg of the recombinant protein mixed with an equal volume of 5 mg of aluminum hydroxide (Imject™ Alum Adjuvant, Thermo Fisher Scientific) (**Figure 1A**). The formulations were kept at 4°C until use.

#### 2.12.1 Intranasal and combined subcutaneous/intranasal route evaluation

Six-week-old, specific pathogen-free (SPF), weaned male and female Vietnamese minipigs were employed. Four groups were included in the experiment (n=4-6/group). Each group received the following immunization protocol: Group 1: adjuvant only, group 2: subcutaneous immunization (SC) only, group 3: intranasal inoculation (IN) only, and group 4: combined immunization (SC/IN), where the first and second doses were SC and the last two were IN. All animals were immunized four times on days 0, 7, 14, and 42 through either SC or IN routes, depending on the group (**Figure 1A**). At the end of the experiments, animals were humanely euthanized, and bronchoalveolar lavages (BAL) from each animal were obtained as detailed previously (45).

### 2.13 Mucosal antibody quantification

Nasal mucus, saliva and blood samples were obtained at days 0, 14, 28, 42, and 56 of the immunization schemes. These samples were collected and processed as detailed previously (27) and stored at -20°C until analysis. Serum and mucosal antigen-binding IgG and IgA were measured by a quantitative ELISA adapted from our Mexican patent and published elsewhere (27, 28).

### 2.14 Microneutralization assays

Mucosal secretions and sera collected on day 56 were analyzed for neutralization activity using the commercial SARS-CoV-2 Surrogate Virus Neutralization Test (sVNT) (GenScript Biotech, Piscataway, NJ, USA) according to the manufacturer’s recommendations. Two COVID-19 convalescent human sera were employed as positive controls. The results obtained were analyzed using the following formula: (1-(absorbance of sample/absorbance positive)) *100. Samples with 30% or more inhibition were considered positive.

### 2.15 Statistical analyses

All data were analyzed and plotted in GraphPad Prism, version 8.0.1 (GraphPad Software, San Diego, California USA, www.graphpad.com), following two-ways analysis of variance (ANOVA) and *post-hoc* unpaired Tukey-Kramer´s, Sidak´s and Mann-Whitney tests for multiple comparisons. Tests were selected according to the type of experiment and are indicated in the figures’ footnotes. An alpha value of 0.05 was set for all analyses.

## 3 Results

### 3.1 Generation of the chimeric gene and heterologous protein expression

Six coding sequences in the RBD domain of the S protein were selected to construct the chimera gene. Five peptides (1, 3–6) contained predicted B-cell epitopes. Peptide 2 contained only predicted T-cell epitopes. Peptides 1, 2, and 3, contained MHC class II-bound T-cell epitopes and all six peptides contained MHC class I-bound T-cell epitopes. The amino acid sequences of each peptide are shown in **Figure 1B**. Peptides were ordered in different positions through a single sequence with or without spacers. More than twenty versions of chimeric proteins were analyzed, but version 17 was selected because it did not contain juxtaposed, new unrelated epitopes. Based on this amino acid sequence, a chimeric gene was constructed *in silico* and commercially synthetized (Bio Basic Inc., Ontario, Canada). Proper cloning of the chimeric gene in pENTR/D-TOPO and p-DEST-17 was confirmed by PCR and sequencing (data not shown). Additionally, the start codon was located, and a proper open reading frame was upheld during cloning. The predicted amino acid sequence was obtained, which confirmed that the chimeric gene was cloned successfully into the expression vector.

The results of the expression experiment are shown in **Figure 1C**. A distinct electrophoretic pattern was observed in the range of 37 kDa to 50 kDa in the insoluble fraction of the induced *E. coli* lysate in lane 4 compared to the noninduced fraction in lane 7. This pattern corresponded to the expected molecular mass of the chimeric protein (48 kDa). These electrophoretic pattern differences were observed only in the insoluble protein fraction of all the analyzed clones (data not shown), while no differences in protein expression were observed in the soluble fractions, indicating that the recombinant protein was expressed, and that it was localized in the insoluble fraction of the lysate (**Figure 1C**, lanes 2 and 5, and lanes 3 and 6). We named this recombinant, multiepitopic, chimera protein CHIVAX.

The antigenicity of the chimeric protein was assessed using Western blot analysis, as illustrated in **Figure 1D**. Chemiluminescence signals were observed when the recombinant protein was incubated with the serum of a recovered COVID-19 individual as well as with a monoclonal antibody specific for the histidine tag. The chemiluminescent signal detected a band, approximately 48 kDa in size, which corresponded to the expected molecular weight of the chimeric protein. This concurrence in protein detection using both antibodies indicates that the same protein is being targeted, further supporting its antigenic properties, especially the presence of epitopes detected by antibodies of recovered individuals and by a commercial monoclonal antibody targeting the tag.

The protein was purified by affinity chromatography using a nickel column, and was eluted in 8 fractions, as shown in **Figure 1E**, where the detection of a single band at 48 kDa was observed in all dialyzed fractions. Among these, fractions three to six had a protein concentration of 1 mg/mL and were selected. They were kept at -80°C and used for the immunization experiments.

### 3.2 Serum antibodies are produced in pigs immunized subcutaneously in a dose-dependent manner

**Figure 2:**
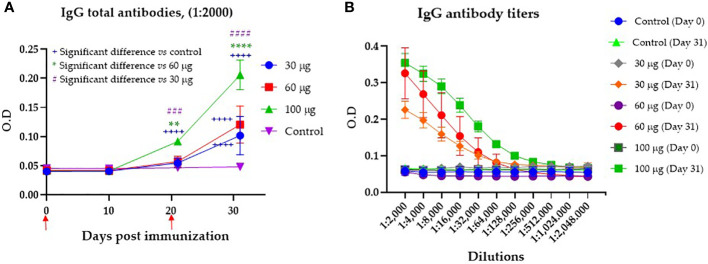
Serum antibody production in pigs immunized with CHIVAX 17.4 by the subcutaneous route. **(A)** Effect of the immunization dose on the production of specific IgG antibodies to the chimeric protein (samples diluted 1:2000). The animals were immunized two times (red arrows). **(B)** Specific IgG antibody titers to the chimeric protein detected in the serum of pigs 31 days after the first immunization with the chimeric protein in the groups immunized with 30, 60 or 100 µg, and the control group. The results are expressed as the mean ± SEM of five independent experiments in quadruplicates. The blue plus symbol represents the statistical difference with the control group, the green asterisk represents differences with 60 µg, and the number violet symbol differences with 30 µg group. Unpaired t-test with Tukey-Kramer´s comparison. The differences were considered significant at: p ≤ 0.01 for two symbols, p ≤ 0.001 for three symbols, and p ≤ 0.0001 for four symbols.

Evaluation of the humoral immune response against the recombinant protein was first assayed in pigs with an immunization scheme consisting of two subcutaneous immunizations on days 0 and 21 with Montanide ISA 71 vg as adjuvant and a final evaluation on day 31. The serum of the pigs from all groups was analyzed by indirect ELISA for the reactivity of IgG antibodies against CHIVAX. **Figure 2A** shows specific IgG antibody levels over the days according to the dose used in each immunized group. All groups of pigs immunized with CHIVAX generated specific IgG antibodies by day 31. However, antibodies started to be detected by day 10 after the initial immunization, and by day 21, the IgG levels of animals immunized with a dose of 100 µg were significantly different from the levels of IgG antibodies detected the same day in the serum of animals immunized with 60 µg (0.057), 30 µg (0.051) and the control group (0.046). On day 31, on the other hand, after a full immunization scheme, it was observed that the total amount of specific antibodies in the group immunized with 100 µg was significantly different (α=0.05) from the total amount of specific antibodies detected on the same day in the serum of animals immunized with 60 µg, 30 µg and the control group (**Figure 2A**). In addition, it was observed that the amount of specific antibodies to the chimeric protein in the serum of animals immunized with 30 or 60 µg on day 31 after immunization was also significantly different (α=0.05) from the control group (**Figure 2A**). The control group, immunized only with adjuvant, never showed any level of specific IgG antibodies during the experiment. **Figure 2B** shows IgG antibody titers at 31 days post immunization of up to 1:256,000 for pigs immunized with 100 μg, IgG antibodies of up to 1:128,000 for the group immunized with 60 μg, and titers of 1:64,000 for the group immunized with 30 μg. None of the preimmunization sera or the sera from pigs immunized only with adjuvant contained any antibody titers.

### 3.3 Immunized pigs generate antibodies specific for individual peptides in the multiepitope vaccine

**Figure 3:**
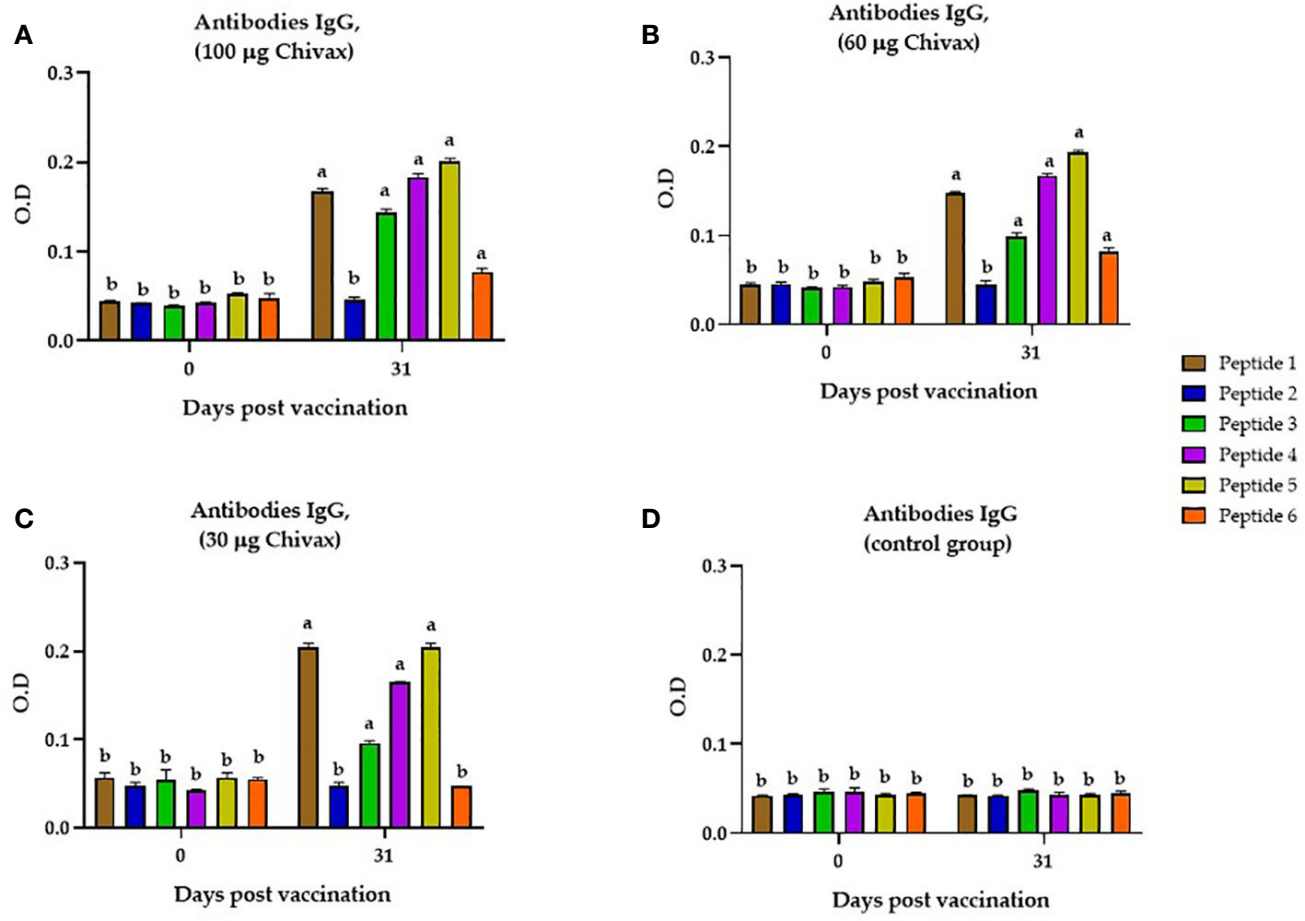
Recognition of individual peptides by the sera of immunized pigs. **(A)** Individual peptide recognition by the serum of animals immunized with 100 µg of CHIVAX protein. **(B)** Individual peptide recognition by the serum of animals immunized with 60 µg. **(C)** Individual peptide recognition by the serum of animals immunized with 30 µg. **(D)** Detection of individual peptides by the serum of animals in the control group, Dilution of serum in all graphs is 1:80. The results are expressed as the mean ± SEM of five independent experiments in quadruplicate. Small letters express significant differences between all the samples with letters different (*p* ≤ 0.01) by Unpaired t-test with Sidak´s multiple comparisons.

To determine whether the multiepitope chimeric protein induced IgG antibodies in the sera from immunized pigs that recognized each individual peptide, an indirect ELISA was performed by coating the plate with each individual peptide and incubating them with sera from pigs in each of the three groups immunized with the recombinant protein (30, 60 and 100 µg). In **Figure 3A**, we observed that the pigs immunized with 100 µg of CHIVAX generated IgG antibodies for peptides 1, 3, 4, 5 and 6 (p<0.05) on day 31, with a significant difference from day 0. Animals immunized with 60 µg (**Figure 3B**) of recombinant protein also produced specific IgG antibodies against peptides 1, 3 4, 5 and 6 (p<0.05). In the group of pigs immunized with 30 µg, the presence of antibodies specific for peptides 1, 3, 4 and 5 was detected. No IgG antibodies against peptide 6 were observed in the immunized animals. No IgG antibodies against peptide 2 were observed in any of the groups (**Figures 3A–C**). No antibody response was detected in the group immunized only with adjuvant as control (**Figure 3D**). No significant difference in peptide-specific antibodies was observed between the sera of animals immunized with different concentrations.

### 3.4 Sera of immunized pigs can block the RBD-ACE2 binding of four VOCs and the ancestral strain

**Figure 4:**
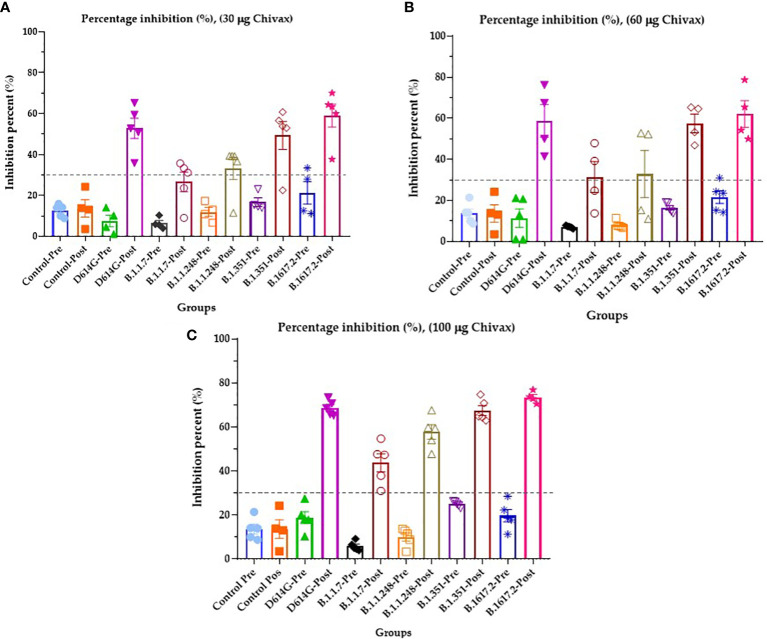
Sera of immunized pigs block the RBD-ACE2 binding of four VOCs and the ancestral strain of SARS-CoV-2. **(A)** The percentage inhibition of RBD-ACE2 binding of serum from pigs immunized with 30 µg of recombinant protein. **(B)** The percentage inhibition of RBD-ACE2 binding of serum from pigs immunized with 60 µg of recombinant protein, and **(C)** The percentage inhibition of RBD-ACE2 binding of serum from pigs immunized with 100 µg of recombinant protein. All graphs represent inhibition percentage (%) against Wuhan-1 (D614G), Alpha (B.1.1.7), Gamma (B.1.1.248), Beta (B.1.351), and Delta (B.1617.2) variants determined at 450 nm. The results are expressed as the mean ± SEM of five independent experiments in triplicate. Each experiment corresponds to an animal immunized with 30, 60, 100 µg of vaccines for duplicated. Each symbol represents an individual. The threshold was 30% inhibition. Modified from 44.

The presence of antibodies in the sera of immunized pigs with the capacity to block RBD-ACE2 binding was determined by a surrogate neutralization assay. For this, the plates were coated with five different recombinant RBDs of SARS-CoV-2: Alpha B.1.1.7, United Kingdom; Beta B.1.351, South Africa; Delta B.1617.2, India; Wuhan-1 D614G, Gamma B.1.1.248, Amazon. The results are shown in **Figure 4**. Animals immunized with 100 µg of the recombinant protein produced neutralizing antibodies against the original Wuhan strain of SARS-CoV-2 with 68.68% inhibition, against the alpha variant with 43.40% inhibition, 57.68% inhibition against the gamma variant, and 67.50% and 73.46% inhibition against the beta and delta variants, respectively. All the preimmune sera were below the threshold value of 30% inhibition (**Figure 4A**). Pigs immunized with 60 µg showed inhibition above 50% against Wuhan, Beta, and Delta Variants (**Figure 4B**), while for Alpha and Gamma variants, 31.43% and 32.84% inhibition were achieved, respectively. Finally, animals immunized with 30 µg of protein produced neutralizing antibodies against the Wuhan variant of SARS-CoV-2 with 52.79% inhibition, against the Beta variant with 49.32% inhibition and against the Delta variant with 59.05% inhibition. For the gamma variant, the inhibition was 33.22%. All the preimmune serum and postimmune serum alpha variants (26.58%) were below the threshold value of 30% inhibition (**Figure 4C**).

### 3.5 Pigs produce antigen-specific IgA and IgG antibodies in nasal, oral, and bronchoalveolar mucosae after subcutaneous and combined immunization schemes

**Figure 5:**
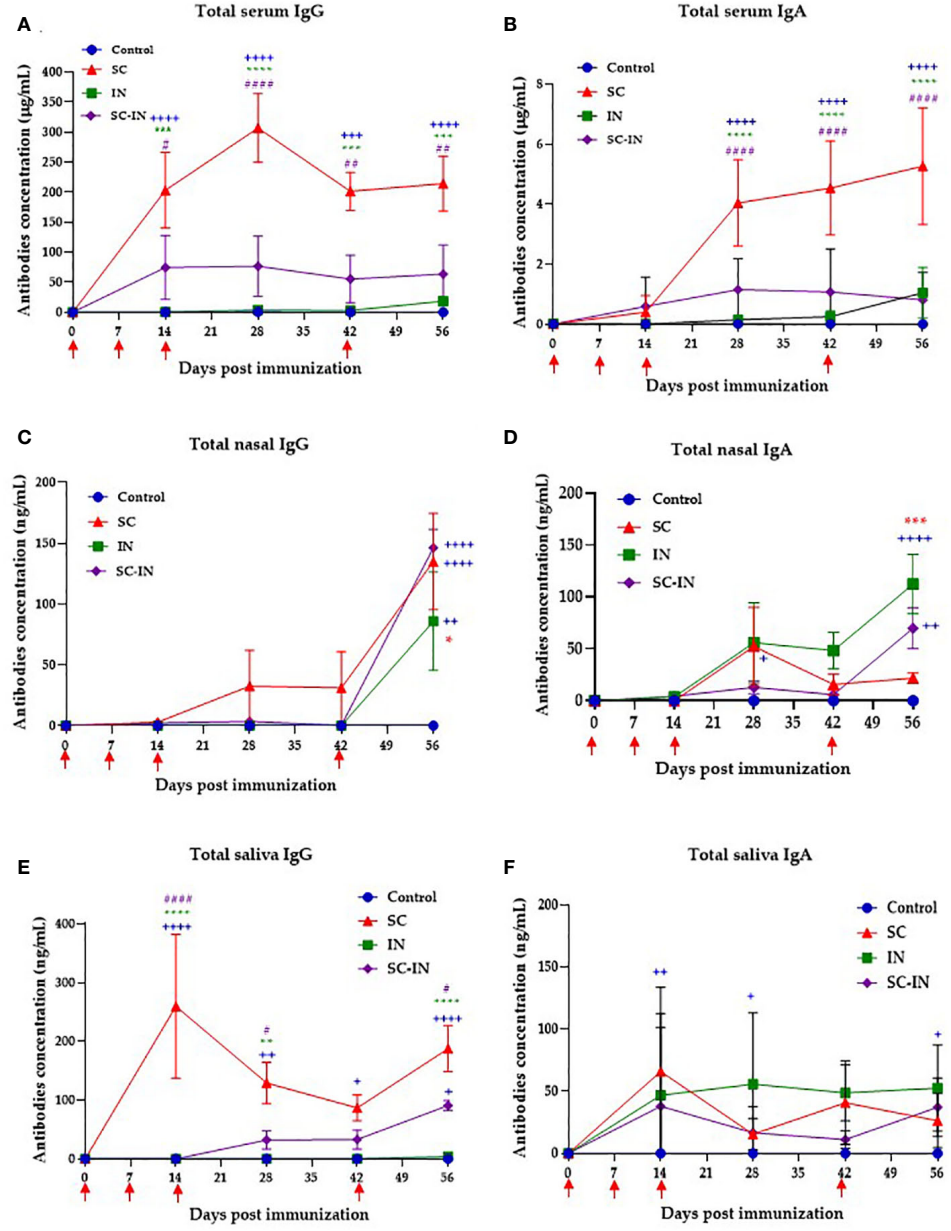
Serum and mucosal IgG and IgA antibody responses in vaccinated minipigs. The animals were immunized at days 0, 7, 14 and 42 by the subcutaneous (SC, red triangles), intranasal (IN, green squares), or a combined SC (days 0 and 7)/IN (days 21 and 42) (purple diamonds) route, and their IgG and IgA concentration were determined by a quantitative ELISA. Serum IgG **(A)**, and IgA **(B)** antibodies. Nasal IgG **(C)**, and IgA **(D)** antibodies. Saliva IgG **(E)**, and IgA **(F)** antibodies. Each symbol is the mean value of 4-6 animals ± SEM. Statistical significance was tested by paired multiple t-tests with the Tukey method. The blue plus symbol represents the statistical difference with the control group, the green and red asterisks represent differences between the IN and SC groups respectively, and the number violet symbol differences with the SC/IN group. The level of significance is as follows: One symbol p ≤ 0.05, two symbols p ≤ 0.01, three symbols p ≤ 0.001, and four symbols p ≤ 0.0001. Unpaired t-test with Mann-Whitney comparison. Scales are different in the graphs.

To determine if the intranasal or combined subcutaneous/intranasal scheme induces antibodies in immunized pigs, we evaluated the presence of specific IgG and IgA antibodies in the mucosae and serum. The results are shown in **Figure 5**, and pigs immunized SC generated specific IgG antibodies in serum, producing a maximum concentration of 300 μg/mL at day 28 (**Figure 5A**). In this group, the IgG response was evident after the second SC immunization producing 203 μg/mL on day 14, and that level was maintained from day 42 until the end of the experiment on day 56. The serum IgG in the group immunized with the combined SC/IN route also appeared on day 14, where it reached the maximum level of 60 μg/mL, and the production was steadily maintained until the end of the experiment. The IN route did not induce IgG levels at any of the times evaluated (**Figure 5A**).

For the serum IgA production, the response arose after the third inoculation in the SC group with a peak of 5 ug/mL on day 56 (**Figure 5B**). In the groups immunized with the IN and the combined SC/IN protocol IgA had no statistical differences from the control group (**Figure 5B**). The IgG levels in nasal mucus increased significantly for all groups after the fourth immunization on day 42, as shown in **Figure 5C**. By day 56, the levels were over 130, 146, and 90 ng/mL for the SC, SC-IN, and IN groups, respectively. For mucous IgA, the IN group showed a significant response (112.4 ng/mL) by day 56 with respect to the control and SC groups. Pigs immunized via SC/IN showed a response of 70 ng/mL with a significant difference between the control group and 25-30 ng/mL for the SC group (**Figure 5D**). For saliva IgG antibodies were early detected (d14) in the SC group, maintaining levels between 100-200 ng/mL throughout the experiment and being significantly superior to the SC/IN group in which saliva IgG level only significantly increased (90.7 ng/mL) by day 56 after the second intranasal boost. The control and IN groups did not show any level of saliva IgG (**Figure 5E**). Finally, for saliva IgA, the three immunized groups produced between 25-60 ng/mL until day 56, with no statistical differences between them and the control group (**Figure 5F**).

**Figure 6:**
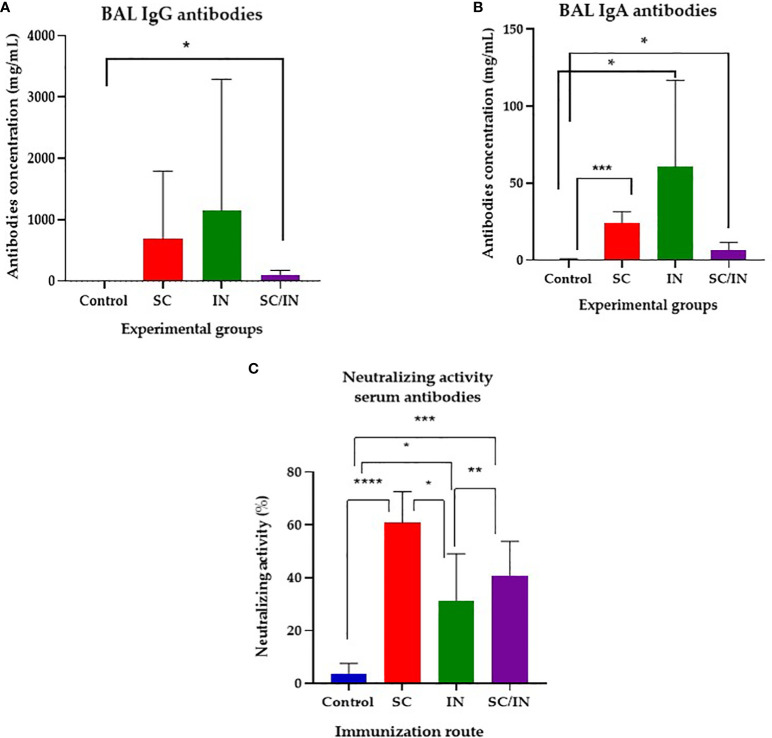
Bronchioalveolar lavage IgG and IgA anti CHIVAX response in immunized pigs, and neutralizing activity evaluation. Pigs were immunized on days 0, 7, 14 and 42 by the subcutaneous (SC), intranasal (IN), or a combined SC (days 0 and 7)/IN (days 21 and 42) route, and their IgG **(A)** and IgA **(B)** concentration were determined by a quantitative ELISA in serum samples from day 56 after the first immunization. The horizontal bar is the mean, and each symbol represents one animal. The scales of the graphs are different. Statistical significance: *p≤0.05, **p≤0.01. Unpaired t-test with Mann-Whitney comparison. **(C)** Anti SARS-CoV-2 neutralizing antibody activity in serum of vaccinated minipigs. Samples were taken at day 56 after four immunizations by the SC, IN, or a combined SC/IN route, and their neutralizing activity was tested using the SARS-CoV-2 Surrogate Virus Neutralization Test (sVNT). Statistical significance was tested by an unpaired t-Student test with Mann-Whitney comparison. *p ≤ 0.05, **p ≤ 0.01, ***p ≤ 0.001, ****p ≤ 0.0001.

In bronchioalveolar lavages (**Figure 6**), taken 14 days after the last immunization, the anti-CHIVAX IgG (**Figure 6A**) and IgA (**Figure 6B**) responses were demonstrated in both SC and SC/IN routes with significant increases over the control group.

### 3.6 Intranasal or combined SC/IN immunization induces neutralizing antibodies in the serum of immunized pigs

Finally, serum antibodies of all immunized groups showed neutralizing activity evaluated using a surrogate virus neutralization assay (**Figure 6C**), with the SC group having the highest activity (up to 60%), followed by the SC/IN group (up to 40%) and the IN group (approximately 30%). There was a significant difference between the SC (≤ 0.0001), IN (≤ 0.05), and SC/IN (≤ 0.001) groups compared with the control group. Among the immunized groups statistical differences between the SC (highest) and the IN, and SC/IN groups were also demonstrated. Mucosal antibodies did not show any neutralizing activity (data not shown).

## 4 Discussion

In this study, we successfully generated a chimeric gene comprising six coding peptides containing predicted B- and T-cell epitopes from the RBD of the S protein of SARS-CoV-2. The resulting chimera was synthesized *in silico*, cloned into expression vectors, and confirmed by PCR and sequencing. When expressed in *E. coli*, a distinct electrophoretic pattern was observed in the insoluble protein fraction, indicative of successful heterologous protein production, with the produced chimeric protein having a molecular mass of approximately 48 kDa. Analysis of its antigenicity through Western blot analysis showed that it was recognized by both the serum of a COVID-19-recovered individual, and a monoclonal antibody specific for the His tag, supporting its antigenic properties and the successful expression of the selected epitopes. The chimeric protein was successfully purified by affinity chromatography, and it was named CHIVAX. There are vaccines for other diseases that were developed using immuno-bioinformatics approaches like the one reported here (46–49). There are vaccines against COVID-19 that use a recombinant protein as the antigen (50, 51) or the RBD of the S protein as the vaccine (52), and they are in clinical trials, supporting their use and success. There are no vaccines for COVID-19 based on a chimeric, multiepitope approach targeting B- and T-cell epitopes of the S RBD; therefore, CHIVAX intends to complement the distinct approaches for vaccine development.

The results demonstrate a significant and dose-dependent humoral immune response to the recombinant chimeric protein in pigs following subcutaneous immunization on days 0 and a booster on day 21 and a final evaluation on day 31. In all groups, specific IgG antibodies were generated by day 31, indicating successful priming of the immune system, as has been shown for similar immunization protocols (53–56), supporting the hypothesis that a chimera recombinant protein containing RBD-specific peptides is immunogenic. However, the quantity of specific IgG antibodies was dose-dependent with pigs receiving 100 µg of the protein showing the highest antibody levels followed by the 60 µg and 30 µg groups, as observed in other immunization protocols for other pathogens (57, 58). The control group, immunized only with the adjuvant, did not produce any specific antibodies, emphasizing the role of the chimeric protein in inducing a humoral immune response. It has been shown that a humoral immune response is necessary for protection against COVID-19 (59, 60). It would be desirable to follow the antibody production for more than 31 days, which was the period evaluated here, and which gives an idea of the time frame of the immune response in vaccinated individuals.

For a chimeric antigen to be advantageous over single peptide vaccines, it must generate an immune response to each of its conforming peptides. To address whether the multiepitope chimeric protein stimulates the production of antibodies that can recognize each individual peptide, we performed an indirect ELISA experiment in which plates were coated with each of the six peptides and incubated with sera from pigs immunized with the recombinant protein. IgG antibodies specific to peptides 1, 3, 4, 5, and 6 were detected in the groups receiving the 100 µg and 60 µg doses, whereas the group that received the 30-µg dose showed antibodies specific for peptides 1, 3, 4, and 5. Notably, peptide 2 did not induce antibodies detected by iELISA in any of the groups. However, this peptide was selected because it contained predicted T-cell epitopes but not B-cell epitopes; therefore, the lack of humoral immune response was somehow expected. In this work, we did not evaluate the cellular immune response of the vaccinated pigs to confirm the presence of a T-cell epitope in peptide 2, nor any of the other peptides, and we did not determine if this antigen induced a CTL response, which is important against viral infections. We showed that the five peptides containing predicted B-cell epitopes did indeed induce antibodies. This suggests that the multiepitope chimeric protein is capable of inducing an antibody response against multiple peptides and that the response against certain peptides may be dose-dependent or vary based on the characteristics of the individual peptide. A similar approach was reported where peptides containing B- and T-cell epitopes were included, but they were administered as a peptide mixture, not built as one single polypeptide chain, even though an immune response was elicited (53). The findings that the antibodies in the sera of pigs immunized with the recombinant protein could effectively block the RBD-ACE2 binding of not only the ancestral SARS-CoV-2 strain (Wuhan-1 D614G) but also four Variants of Concern (VOC): Alpha B.1.1.7, Beta B.1.351, Gamma B.1.248, and Delta B.1617.2 support our strategy of designing chimeric antigens with conserved sequences in the RBD of the Spike protein in multiple strains without the need to use a eukaryotic system, demonstrating that linear peptides, without glycosylation, induce neutralizing antibodies. These results together support our approach that identifying biologically functional and conserved epitopes based on immuno-bioinformatics induces the production of neutralizing antibodies, which has been confirmed by our group several times (9–11, 61, 62). Thus, these results demonstrate that the multiepitope chimeric protein could potentially provide protection against the SARS-CoV-2 VOC evaluated here, albeit the neutralizing capacity appears to be dose-dependent and varies among the different viral strains, as shown here and in other works (63, 64). In this work, we did not test neutralization against any omicron subvariants, which appeared after we performed the experiments. However, this work highlights the design of a chimeric multiepitope recombinant protein as a strategy to circumvent the problem of new variants escaping the immune response. By incorporating conserved epitopes in the RBD of four VOC and the ancestral strain, we have shown the production of antibodies that neutralize all of them, even though the chimera protein developed here does not induce antibodies targeting conformational epitopes. This strategy can now be pursued by incorporating conserved peptides in the RBD of the Omicron subvariants, which are the predominant VOC in the world (65, 66).

On the other hand, mucosal immunity is of paramount importance to prevent and control infectious diseases (67). We compared parenteral (SC) and mucosal (IN) immunization to induce systemic and mucosal immune response and, seeking to avoid tolerance induction and the uncertainty of the mucosal doses administered, a SC/IN protocol was also tested (27, 28, 45). Our results indicated that both IN and SC immunizations stimulate the production of IgA and IgG antibodies, with specific response dynamics and locations (**Figure 5**). For serum, significant production of IgG was observed after the second SC dose, whereas the IgA response was noticeable after the third inoculation. Interestingly, nasal mucosae demonstrated a robust IgG anamnestic response after the second boost in all groups, and the IgA response was significant in the IN group by day 42, indicating effective mucosal priming and memory response. Saliva IgG levels appeared early and were maintained in the SC group, suggesting that this route of immunization has systemic effects, which impacts saliva antibodies, whereas the SC/IN group showed a significant rise in saliva IgG levels after the second IN boost. In this work we used the recombinant protein emulsified with aluminum hydroxide for the subcutaneous (SC) immunization with relatively small dose of antigen. For the intranasal (IN) immunization we used the antigen alone. Using this strategy, significant increases in saliva IgA were observed only in the IN route, but all groups showed high individual variation, as expected. In bronchoalveolar lavage (BAL) samples, taken on day 56 (**Figures 6A, B**) the IgG and IgA anti-CHIVAX responses were significantly higher with the SC routes, suggesting that the direct application of the Ag to the mucosa (IN), may not be efficient enough to prime the lung immune system. Finally, the virus neutralization activity was demonstrated in serum samples at day 56 post-immunization (**Figure 6C**) from the three experimental groups. Among methods of immunization, the SC route showed superior neutralizing activity than the other two. None of the mucosal samples showed this activity, probably due to the low level of antibodies at that day, which give room to test other doses of immunogen to increase the mucosal response.

These results demonstrate that the multiepitope chimeric protein can elicit local mucosal and systemic immune responses, depending on the route of immunization. The detection of IgA and IgG antibodies in mucosal samples supports the potential for both systemic and mucosal immunity, which may be critical for preventing both infection and transmission of SARS-CoV-2 (65, 66, 68, 69). Further adjustments to the antigen/adjuvant ratio may improve the efficacy of the immunization protocols.

## 5 Conclusion

In conclusion, this study effectively demonstrates the generation and immunogenic properties of a multiepitope chimeric protein engineered to include six coding sequences from the RBD domain of the SARS-CoV-2 S protein. The elicited antibodies were capable of recognizing individual epitopes on the chimeric protein and demonstrated the capacity to block RBD-ACE2 binding of the ancestral SARS-CoV-2 strain and four VOCs. The immunization approach also stimulated IgA and IgG antibody production in both systemic and mucosal compartments.

These findings underscore the potential of this multiepitope chimeric protein as a promising candidate for a broadly protective SARS-CoV-2 vaccine and the use of alternative routes for antigen administration to induce systemic and local immunity. Additionally, the results highlight the importance of the pig is a suitable translational model for pre-clinical studies on human vaccine evaluation and the need for further research into dose, route of administration, and immune response characteristics to optimize vaccine design and administration strategy.

## Data availability statement

The original contributions presented in the study are included in the article/supplementary material, further inquiries can be directed to the corresponding authors.

## Ethics statement

The studies involving humans were approved by Comité de ética. Universidad Autónoma de Querétaro. The studies were conducted in accordance with the local legislation and institutional requirements. The participants provided their written informed consent to participate in this study. The animal studies were approved by Comité de ética de la Universidad Autónoma de Querétaro. The studies were conducted in accordance with the local legislation and institutional requirements. Written informed consent was obtained from the owners for the participation of their animals in this study.

## Author contributions

JM: Conceptualization, Funding acquisition, Project administration, Supervision, Writing – original draft, Writing – review & editing. DH: Formal analysis, Investigation, Methodology, Visualization, Writing – original draft, Writing – review & editing. MV: Conceptualization, Investigation, Methodology, Project administration, Resources, Supervision, Writing – original draft, Writing – review & editing. LV: Investigation, Methodology, Writing – original draft, Writing – review & editing. RB: Investigation, Methodology, Resources, Writing – review & editing. AV: Formal analysis, Investigation, Methodology, Writing – review & editing. MR: Investigation, Methodology, Writing – review & editing. MF: Investigation, Methodology, Writing – review & editing. CP: Formal analysis, Investigation, Methodology, Writing – review & editing. JH: Investigation, Supervision, Writing – review & editing. EM: Investigation, Methodology, Writing – review & editing. DH: Investigation, Methodology, Writing – review & editing. MM: Investigation, Methodology, Writing – review & editing. AM: Investigation, Methodology, Writing – review & editing. CR: Investigation, Methodology, Writing – review & editing. TG: Funding acquisition, Resources, Supervision, Writing – review & editing.
